# Nitrogen and phosphorous dynamics with stand development of *Pinus massoniana* plantations in Southeast China

**DOI:** 10.3389/fpls.2023.1139945

**Published:** 2023-03-24

**Authors:** Wen Guo, Pengyu Jiao, M. Larry Lopez C, Zelong Chen, Lili Wei, Xian Liu, Yalin Hu, Yuzhe Wang

**Affiliations:** ^1^ College of Forestry, Fujian Agriculture and Forestry University, Fuzhou, China; ^2^ Faculty of Agriculture, Yamagata University, Tsuruoka, Japan; ^3^ Institute of Urban Environment, Chinese Academy of Sciences, Xiamen, China; ^4^ University of Chinese Academy of Sciences, Beijing, China

**Keywords:** *Pinus massoniana*, nutrient use strategy, resorption efficiency, resorption proficiency, phosphorus, nitrogen

## Abstract

**Introduction:**

Nutrient resorption is a key mechanism to conserve nutrients and overcome nutrient limitation in perennial plants. As an important afforested tree species in subtropical regions, *Pinus massoniana* grows well in nutrient-poor environments, however, the age-related pattern of nutrient acquisition strategy and the underlying mechanisms in *P. massoniana* plantations remain unclear.

**Methods:**

In this study, concentrations of nitrogen (N) and phosphorus (P) were measured in green and senesced needles, roots and soil samples collected from *P. massoniana* plantations with different stand ages (9-, 17-, 26-, 34- and 43-year-old) in south China. From these samples, nutrient resorption efficiency (RE) and stoichiometry were calculated.

**Results:**

Needle PRE significantly decreased with stand age, while there was no clear pattern of NRE along the stand development. Green needle N:P in older stands was significantly lower than in younger ones. Senesced needle C:P and N:P significantly decreased with stand age. Root and soil available P concentrations were significantly higher in older stands than in younger ones, and PRE was negatively correlated with soil available P concentration.

**Discussion:**

There was a shift from “conservative consumption” to “resource spending” P-use strategy, and P limitation decreased with stand development of *P. massoniana* plantations. The results provide information of changes in nutrients dynamics, which is relevant for the sustainable management of subtropical forest plantations.

## Introduction

Nutrient limitation of plant growth and productivity is widespread in global terrestrial ecosystems (LeBauer and Treseder, 2008; Hou et al., 2020). Root uptake and leaf nutrient resorption are two main mechanisms used by plants to acquire and conserve nutrients, respectively (Cleveland et al., 2013; Brant and Chen, 2015; Seidel et al., 2022). Nutrient resorption from senescing leaves is an important nutrient conservation strategy for perennial plants (Aerts, 1996). They are translocated to other living plant tissues before leaf abscission and reused for plant growth and biomass production (Hill, 1980), which sustains the productivity of forest ecosystems (Yuan and Chen, 2015). This is especially important in nutrient-limited ecosystems, where nutrient resorption could make plants rely less on external soil nutrient supply (Kobe et al., 2005). Aerts (1996) showed that about 50% of nitrogen (N) and 52% of phosphorus (P) in green leaf were resorbed by global perennial plants, and they can contribute 31% to 40% of the annual plant N and P demand, respectively (Cleveland et al., 2013; Seidel et al., 2022). Although previous studies focus mainly on nutrient resorption from leaves, a comparable amount of nutrients can also be recycled *via* resorption by other plant organs such as root, twig and stem (Brant and Chen, 2015; Chen et al., 2015; Veneklaas, 2022). Nutrient resorption is generally determined by calculating the differences of nutrient content between green and senesced leaves (nutrient resorption efficiency, NuRE) (Vergutz et al., 2012). Nutrient resorption can also be estimated by the nutrient content reduction in senesced leaves, namely nutrient resorption proficiency (NuRP), which influences litter quality, decomposition and nutrient cycling (Killingbeck, 1996; Aerts and Chapin, 2000; Wardle et al., 2009). Therefore, NuRE and NuRP are considered useful indicators to detect potential nutrient limitation for plant growth.

At the global scale, nutrient resorption is influenced by climate and environmental conditions (Yuan and Chen, 2009; Vergutz et al., 2012; Mediavilla et al., 2014), although the responses of NuRE to climate are also related to soil nutrient availability. At the local scale, nutrient resorption is mainly determined by plant nutrient demand and soil nutrient availability (Luyssaert et al., 2005; Tully et al., 2013; Brant and Chen, 2015; Wu et al., 2020). Plant growth rate, nutrient demand and soil nutrient availability varied with increasing stand age (England and Attiwill, 2006; Yuan and Chen, 2010; Kuznetsova et al., 2011; Chen et al., 2016; Wu et al., 2020), which might result in age-related variations of nutrient resorption patterns. However, in previous studies, where stand age was considered, there were no consistent patterns of NRE, as shown by Sun et al. (2016) and Yan et al. (2018) who found increases, while Wu et al. (2020) found a decrease or no significant change was observed as in Chang et al. (2017) and Zhang et al. (2018). In north China, Xu et al. (2020) found that the response of NRE to stand age varied among four main afforested tree species (*Robinia pseudoacacia*, *Pinus tabuliformis*, *Armeniaca sibirica*, and *Caragana korshinskii*) on the Chinese Loess Plateau, which was attributed to the differences in growth types, plant lifespan and N-fixation capacity. Similarly, both age-related increase and decrease in PRE with stand age were observed in previous studies (Yan et al., 2018; Wu et al., 2020). Needle PRE consistently increased with the stand development of Masson pine plantations in central China (Ali et al., 2022), while significant increase of Masson pine needle PRE was only observed at the early stage of development in soil erosion areas of southern China (Liu et al., 2016). Thus, responses of NuRE to stand age varied with tree species, nutrient elements and soil nutrient availability.

Soil nutrient status may also influence leaf nutrient resorption (Tully et al., 2013). In high-nutrient environment, soil-driven nutrients are less expensive to acquire than those from resorption, and plants would favor root capture than resorption (Wright and Westoby, 2003). Thus, plants growing in nutrient-poor soils generally displayed higher NRE in comparison to those from nutrient-rich environments (Kobe et al., 2005; Reed et al., 2012). However, it is not always the case that plants enhance nutrient resorption in low-fertility soils (Aerts, 1996). Wu et al. (2020) reported that the NRE was positively correlated with soil N stock in different-aged *Cunninghamia lanceolata* plantations, and no significant relationship between NRE and soil N content was observed across the *Robinia pseudoacacia* plantation chronosequence (Deng et al., 2019). Previous studies showed inconsistent trends of soil nutrients content (especially P) with increasing stand age in forest plantations. For example, soil total and available P decreased with increasing stand age in *Larix kaempferi* plantations in northeast China (Chen et al., 2016), while increased PRE and enhanced P limitation were observed in older larch plantations (Yan et al., 2018). However, increasing soil P availability along with stand development was observed in a Masson pine plantation in southeast China (Liu et al., 2016). Therefore, the increased soil P content in older plantations might reduce the dependence of plant on leaf resorption for nutrient conservation and instead rely more on root uptake. However, the influence of soil nutrient status on age-related resorption strategies of N and P is still not well understood.

*P. massoniana* Lamb. is an important pioneer and afforestation species in south China (Luo et al., 2013), and it has been widely planted for timber production and land restoration (Dou et al., 2013; Liu et al., 2016). Subtropical plantations are generally limited by P due to the low soil P availability, and it appears that it is a shift from N limitation to P limitation during forest stand development (Wu et al., 2020). However, *P. massoniana* can grow well in nutrient-poor even barren areas, showing high adaptability and tolerance to environmental stress. Liu et al. (2016) found increases in soil nutrient (especially P) content with stand age in *P. massoniana* plantations. Whether the age-related variations of soil nutrient availability would result in changes in the nutrient acquisition strategy of *P. massoniana* remains unclear. The objective of this study is to evaluate how nutrient (N and P) resorption strategy and limitation status change during stand development of *P. massoniana* plantations. We hypothesized that: 1) needle P resorption efficiency of *P. massoniana* decreases with increasing stand age, since soil P availability generally increases with stand development of *P. massoniana* plantations, 2) needle NRE is regulated by the interaction of soil N status and its eco-physiological characteristics rather than soil N availability alone, 3) P limitation decreases with increasing stand age in subtropical *P. massoniana* plantations.

## Materials and methods

### Study site

**Figure 1:**
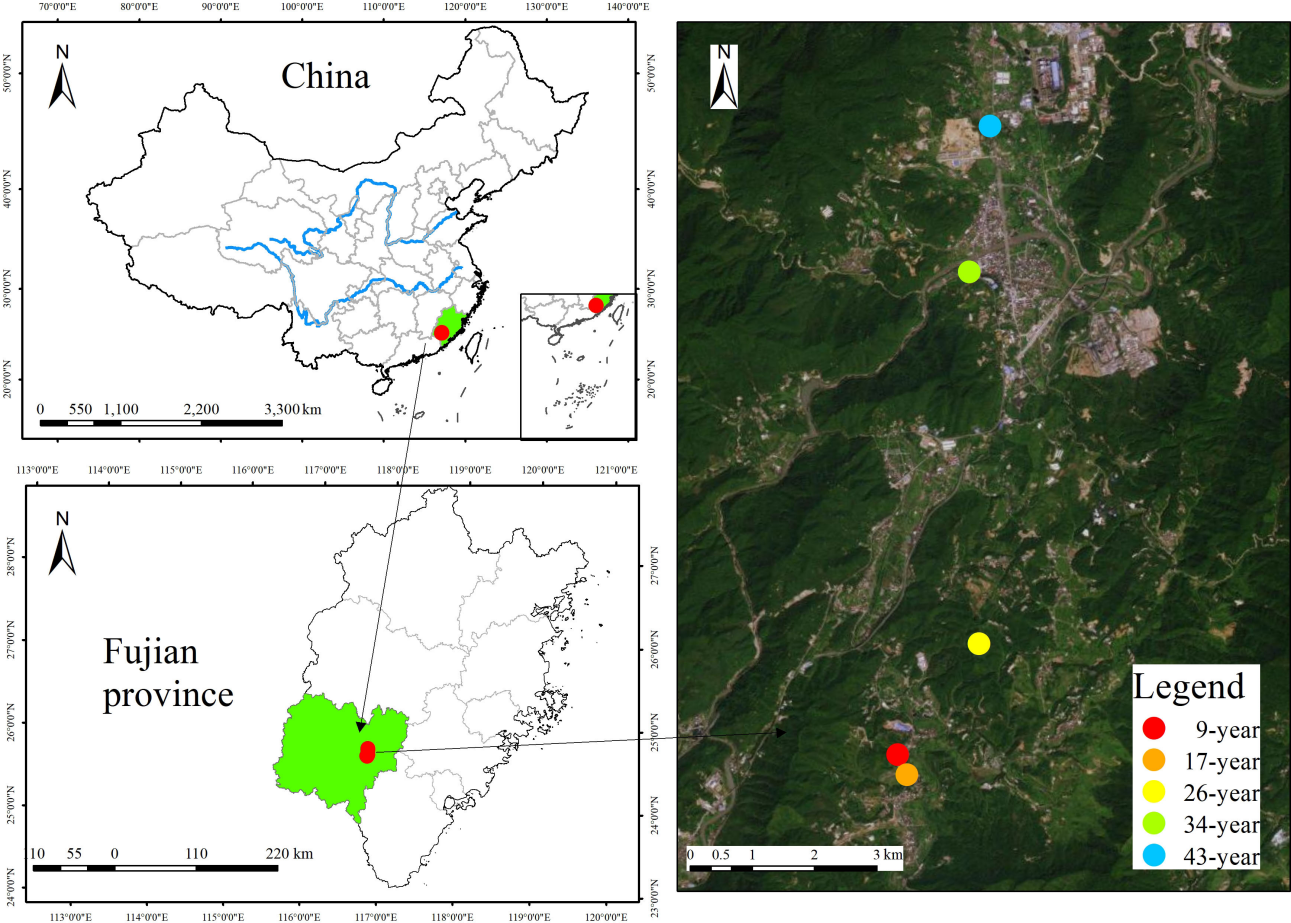
Location of different-aged *Pinus massoniana* plantations in Longyan city, Fujian province, China.

This study was conducted at the Yanshi Forestry Station (25°22′-25°24′ N, 117°13′-117°15′ E, elevation 400-800 m), Longyan city, Fujian province, southeast China (**Figure 1**). The region is characterized by subtropical monsoon climate, with a mean annual temperature of 18.6°C, a mean annual precipitation of 1369 mm, and a frost-free period of 291 day. Soil is developed on granite and classified as Ferric Acrisol according to the World Reference Base soil classification system. Using a space-for-time approach, we selected five even-aged and pure *P. massoniana* plantations (9, 17, 26, 34, and 43-year-old) with similar site conditions (e.g. topography and soil type etc., **Table 1**), and these five age classes represent the juvenile, middle-age, near-mature, mature and over-mature stage of *P. massoniana* stand development, respectively (GB/T 26424-2010, 2011). In each age class, four replicate plots (20 × 20 m) were randomly selected and at least 30 m apart from each other.

**Table 1:** Summary of the *Pinus massoniana* plantations with different ages.

Stand age (year)	DBH (cm)	Height (m)	Density (n/hm^2^)	Altitude (m)	Canopy density	Aspect	Slope (°)
9	7.5	5.3	1880	303	0.30	SW	21
17	11.0	6.5	1568	359	0.55	SE	21
26	15.1	10.7	1050	383	0.40	SW	22
34	17.9	10.7	920	307	0.35	SW	19
43	26.4	18.9	645	316	0.50	SE	14

### Sample collection and chemical analysis

In each plot, based on the diameter at breast height (DBH) and tree height, three healthy trees in each plot were selected for needles collection in October 2020. For each tree, 12 branches were collected by an averruncator in the upper, middle and lower canopy layers from four direction. The current-year (green) needles and needles that were about to fall off, naturally senescent and yellow in color, were collected from the branches and then composed to form one sample per tree, yielding a total number of 60 samples (3 trees×4 plots×5 stand ages) for green and senesced needles, respectively. In each plot, composed soil samples were collected at 5 sampling points after removing litter and humus. Soil samples were sieved in a 2-mm mesh for further analysis. Fine roots were collected following the method described by Pregitzer et al. (2002). Briefly, three standard trees based on the average height and DBH were randomly chosen in each plot. After removing surface litter layer, three soil cores (10 cm length ×10 cm height × 20 cm depth) were collected using a shovel within 1 m distance from the trunk. Fine root segments of *P. massoniana* with the diameter of less than 2 mm were identified and excavated from the soil cores, and soil subsamples were collected as well. Root and soil samples were sealed in plastic bags and stored in an ice box. Both root and soil samples were pooled for each plot, and a total number of 20 soil (root) samples were collected in the five age groups of *P. massoniana* plantations.

All plant samples were dried at 55°C in an oven to a constant mass. Needle and soil total carbon (TC) and total N content was determined by Elementar vario ISOTOPE cube (Germany). Needle and soil total P content were measured by the molybdenum-antimony colorimetric method using a 723A spectrophotometer (AOXI Instrument, Shanghai, China) (Wang et al., 2008). Soil nitrate (NO_3_-−N) and ammonium (NO_4_+−N) content was determined by auto discrete analyzers (SmartChem2000, Italy) after extracted by 2 mol·L^-1^ KCl solution. Total organic carbon analyzer (TOC, Kyoto, Japan) was used to measure the soil dissolved organic C (DOC) and dissolved organic total nitrogen (DTN). Dissolved organic N (DON) was calculated by deducting dissolved inorganic N ( ${\text{NO}}_{3}^{\;-}-\text{N}+{\text{NH}}_{4}^{\;+}-\text{N}$ ) from DTN. Soil available P (AP) was extracted with a 0.5M NaHCO_3_ solution and measured by the molybdenum blue method (Olsen et al., 1954).

### Calculations

Nutrient resorption efficiency (NuRE) was calculated using the following equation:


$$\text{RE}\left(\%\right)=\left(1-\frac{{\text{Nu}}_{\text{senesced}}}{{\text{Nu}}_{\text{green}}}\text{MLCF}\right)\times 100\%$$

Where Nu_senesced_ and Nu_green_ are N and P concentrations in senesced and green needles, respectively. The Mass Loss Correction Factor (MLCF) of 0.745 for coniferous species was used (Vergutz et al., 2012).

### Statistical analyses

All data were checked for normality (Kolmogorov-Smirnov’s test) and homogeneity of variance (Levene’s test) prior to statistical analysis. One-way ANOVA was conducted to test the differences of NuRE, plant tissues and soil nutrient concentrations, as well as associated stoichiometry among the different aged *Pinus massoniana* plantations. Simple linear regression analysis was used to identify the relationship between stand age and NuRE (NRE, PRE, and PRE:NRE). Pearson correlation was conducted to explore the relationships between NuRE and needle, soil total and available nutrient concentrations. All the above mentioned statistical analysis was carried out using SPSS 22.0 for windows (IBM, New York, USA). Redundancy analysis (RDA) was conducted to elucidate the relationship between N and P content (e.g. N and P content of soil, needle and root) and NuRE attributes (PRE, NRE and PRE:NRE). The indicators were excluded when there was co-linearity (VIF>10) in the RDA test. RDA was performed in R v.4.1.0 software with the vegan package (R Development Core Team, 2018). Explanatory rates of individual indicator were calculated using the rdacca.hp package.

## Results

### Soil properties

Soil pH values initially increased with time but then decreased (*p*<0.05), reaching its highest value in the 17-year-old plantation (**Table 2**). Soil moisture, total C, total N, DOC, DON and available P were significantly higher in the 34- and 43-year-old plantations in comparison to the 9-and 17-year-old plantations (*p*<0.05). Soil total P and ${\text{NH}}_{4}^{\;+}-\text{N}$ increased from stand age 7 to 34 and then decreased in the 43-year-old plantation, while ${\text{NO}}_{3}^{\;-}-\text{N}$ steadily increased with increasing stand age (*p*<0.05).

**Table 2:** Soil properties in *Pinus massoniana* plantations with different stand ages.

Soil property	Stand age (year)				
	9	17	26	34	43
pH	4.66 ± 0.02b	6.21 ± 0.05a	4.62 ± 0.02b	4.32 ± 0.05c	4.00 ± 0.03d
Moisture content (%)	13.37 ± 1.2b	14.79 ± 0.45b	10.32 ± 0.47c	21.35 ± 0.65a	19.86 ± 0.63a
Total C (g kg^-1^)	4.86 ± 0.16d	18.54 ± 1.37c	31.2 ± 2.23b	47.7 ± 2.76a	35.64 ± 2.63b
Total N (g kg^-1^)	0.41 ± 0.04d	0.83 ± 0.06c	1.78 ± 0.14b	2.52 ± 0.15a	1.96 ± 0.07b
Total P (g kg^-1^)	0.20 ± 0.02c	0.31 ± 0.04ab	0.26 ± 0.02bc	0.38 ± 0.03a	0.31 ± 0.05ab
${\text{NH}}_{4}^{\;+}-\text{N}$ (mg kg^-1^)	3.25 ± 0.15c	2.77 ± 0.11c	6.69 ± 0.59b	9.81 ± 1.3a	3.36 ± 0.12c
${\text{NO}}_{3}^{\;-}-\text{N}$ (mg kg^-1^)	0.40 ± 0.05d	0.87 ± 0.15d	5.02 ± 0.32c	7.35 ± 0.02b	12.52 ± 0.79a
DOC (mg kg^-1^)	202.78 ± 15.01b	194.87 ± 21.16b	207.25 ± 13.34b	439.99 ± 8.63a	436.02 ± 22.9a
DON (mg kg^-1^)	52.75 ± 1.8b	54.28 ± 0.96b	51.49 ± 1.13b	70.82 ± 4.17a	68.81 ± 1.93a
Available P (mg kg^-1^)	1.26 ± 0.08d	1.61 ± 0.17d	4.19 ± 0.25a	2.60 ± 0.13c	3.54 ± 0.15b
N:P	2.12 ± 0.08b	2.80 ± 0.41b	6.96 ± 0.65a	6.72 ± 0.53a	6.77 ± 1.12a

Data are mean ± stand error (n=4). DOC: dissolved organic carbon, DON: dissolved organic nitrogen. Different lowercase letters indicated significant differences among the stand ages (p<0.05).

### Plant nutrients and stoichiometry

**Figure 2:**
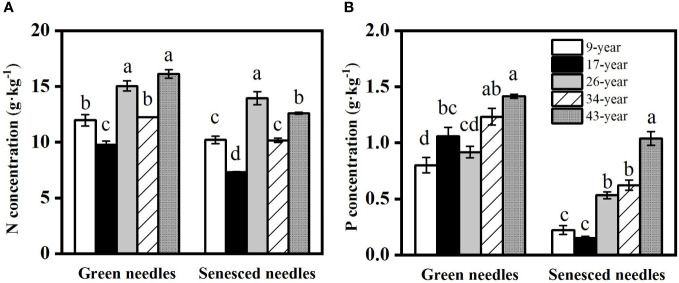
Changes in nitrogen **(A)** and phosphorus **(B)** concentrations in green and senesced needles in *Pinus massoniana* plantations with different stand ages. Different lowercase letters indicated significant differences among the stand ages for each needle type (*p*<0.05).

**Figure 3:**
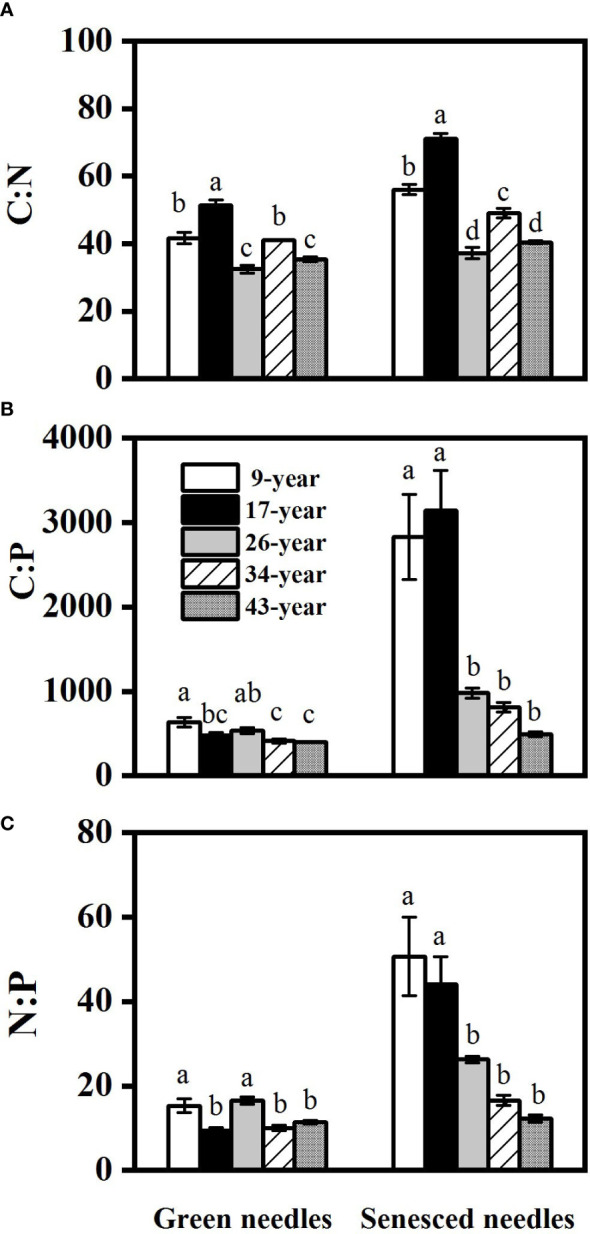
Changes in needle C:N **(A)**, C:P **(B)** and N:P **(C)** in *Pinus massoniana* plantations with different stand ages. Different lowercase letters indicated significant differences among the stand ages for each needle type (*p*<0.05).

**Figure 4:**
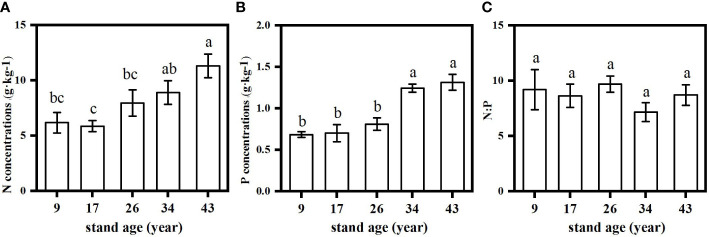
Changes in root N **(A)**, P **(B)** and N:P **(C)** in *Pinus massoniana* plantations with different stand ages. Different lowercase letters indicated significant differences among the stand ages (*p*<0.05).

Both green and senesced needle P concentrations significantly increased with stand age (**Figure 2**), while no regular pattern of N concentrations with stand age was observed, with the lowest N concentration found in the 17-year-old plantation. Green needle N:P values in the 9- and 26-year-old plantations (15.3 and 16.5, respectively) were significantly higher than those in the 17- (9.4), 34- (10.0) and 43-year-old plantations (11.4) (*p*<0.05) (**Figure 3**). Senesced needle C:P and N:P significantly decreased with stand age (*p*<0.05). Root N and P concentration increased with stand age (**Figure 4**), with significant higher concentrations in the 34- and 43-year-old plantations than those in the 9-, 17- and 26-year-old plantations (*p*<0.05). No significant differences of root N:P values were observed among different stand ages (*p*>0.05).

### NRE, PRE and PRE:NRE

**Figure 5:**
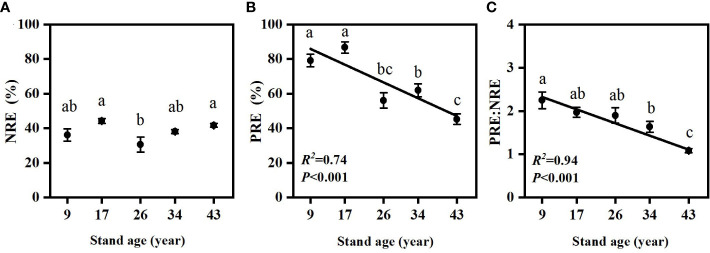
Changes in needle N resorption efficiency **(A)**, needle P resorption efficiency **(B)** and PRE:NRE **(C)** in *Pinus massoniana* plantations with different stand ages. Different lowercase letters indicated significant differences among the stand ages (*p*<0.05).

NRE and PRE were significantly different among stand ages (**Figure 5**). Needle PRE decreased with stand age (*p*<0.01), while needle NRE in 17- and 43-year-old plantations were significantly higher than in the 26-year-old plantation (*p*<0.05). PRE:NRE significantly decreased with increasing stand age (**Figure 5**, *p*<0.001).

### Relationships between needle NuRE and soil nutrient contents

Nitrogen concentration in roots, green and senesced needles were positively correlated with soil total N, N:P and ${\text{NO}}_{3}^{\;-}-\text{N}$ content, and negatively correlated with soil pH (**Table 3**). A significant negative correlation was observed between NRE and N concentration in senesced needles (*p*<0.01). The P concentrations of roots, green and senesced needles were positively correlated with soil water content and N:P (**Table 4**). There was a positive correlation between senesced needle P concentration and soil available P content (*p*<0.01). PRE was negatively correlated with senesced needle P concentration, soil N:P and available P content (*p*<0.001), and a positive relationship was observed between PRE and soil pH (*p*<0.001).

**Table 3:** Pearson correlations between nitrogen resorption efficiency (NRE) and N content in plant and soil.

	Soil pH	SWC	Soil total N	Soil N:P	Soil ${\text{NO}}_{3}^{\;-}$	Soil ${\text{NH}}_{4}^{\;+}$	Soil DON	Green needle N	Senesced needle N
Green needle N	-0.76 (<0.001)	0.07 (0.78)	0.49 (<0.05)	0.61 (<0.01)	0.75 (<0.001)	0.13 (0.58)	0.05 (0.85)	–	0.87 (<0.001)
Senesced needle N	-0.74 (<0.001)	-0.18 (0.45)	0.46 (<0.05)	0.66 (<0.01)	0.56 (<0.05)	0.22 (0.36)	0.21 (0.38)	0.87 (<0.001)	–
Root N	-0.58 (<0.01)	0.38 (0.10)	0.61 (<0.01)	0.69 (<0.001)	0.74 (<0.001)	0.16 (0.50)	0.50 (<0.05)	0.53 (<0.05)	0.61 (<0.001)
NRE	0.30 (0.19)	0.42 (0.07)	-0.12 (0.61)	-0.29 (0.21)	-0.21 (0.37)	0.07 (0.77)	0.19 (0.42)	-0.13 (0.59)	-0.60 (<0.01)

SWC, soil water content; DON, dissolved organic nitrogen.

**Table 4:** Pearson correlations between phosphorus resorption efficiency (PRE) and P content in plant and soil.

	Soil pH	SWC	Soil total P	Soil N:P	Soil AP	Green needle P	Senesced needle P
Green needle P	-0.32 (0.18)	0.70 (<0.001)	0.38 (0.10)	0.47 (<0.05)	0.27 (0.24)	–	0.70 (<0.01)
Senesced needle P	-0.78 (<0.001)	0.50 (<0.05)	0.18 (0.45)	0.79 (<0.001)	0.67 (<0.01)	0.70 (<0.01)	–
Root P	-0.63 (<0.01)	0.71 (<0.001)	0.36 (0.12)	0.68 (<0.001)	0.44 (0.05)	0.70 (<0.01)	0.80 (<0.001)
PRE	0.76 (<0.001)	-0.25 (0.29)	-0.13 (0.60)	-0.82 (<0.001)	-0.82 (<0.001)	-0.40 (0.08)	-0.91 (<0.001)

SWC, soil water content; AP, available P.

RDA results showed that axes 1 and 2 accounted for 59.45% and 10.30% of the overall variance of NuRE attributes. The dominant driving factors of NRE were soil TP (22.39%, *p*<0.05) and green needle P content (11.24%, *p*<0.01), and PRE was significantly influenced by soil AP content (34.51%, *p*<0.01). NRE:PRE was mainly influenced by soil TN (9.30%, *p*<0.01) and green needle P content (36.61%, *p*<0.05). In addition, there was a shift from P resorption to N resorption-dominated pattern with increasing stand age, with the transition occurred in 26- to 34-year-old plantations.

## Discussion

### Changes in nutrient limitation pattern with stand development

N and P are the most common limiting nutrients for plant productivity in terrestrial ecosystems (Elser et al., 2007; Friederike et al., 2016). Green leaf N:P has been considered as a useful indicator for detecting nutrient limitation status of plant growth (Koerselman and Meuleman, 1996; Tessier and Raynal, 2003). Koerselman and Meuleman (1996) suggested that N:P values lower than 14 indicate that plant growth is limited by N, while N:P>16 represents P limitation and N:P values between 14 and 16 indicate that plant growth is co-limited by both N and P. A recent global meta-analysis showed that there is generally a shift from N limitation to P limitation with stand development of forest plantations (Zhang et al., 2022). However, our results showed that the older stands (34- and 43-year-old) and the 17-year-old were limited by N, 26-year-old plantation was limited by P, and 9-year-old plantation was co-limited by N and P. Therefore, needle N:P cannot be used as the only criterion for assessing nutrient limitation (Craine et al., 2008), and accordingly threshold concentration of specific nutrients (20 g kg^−1^ for N and 1 g kg^−1^ for P) has been suggested as an additional indicator of nutrient limitation (Lockaby and Conner, 1999; Zeng et al., 2017). In our study, N limitation was indicated by the consistent lower needle N concentration than the threshold across all stand ages, while P concentration gradually increased from 0.80 g kg^−1^ in the youngest stand (9-year-old) to 1.42 g kg^−1^ in the oldest stand (43-year-old) (**Figure 2**), indicating that P limitation decreased in older stands. Collectively, the decrease of P limitation with stand development of *Pinus massoniana* plantation was supported by both the needle P status and the N:P ratio.

**Figure 6:**
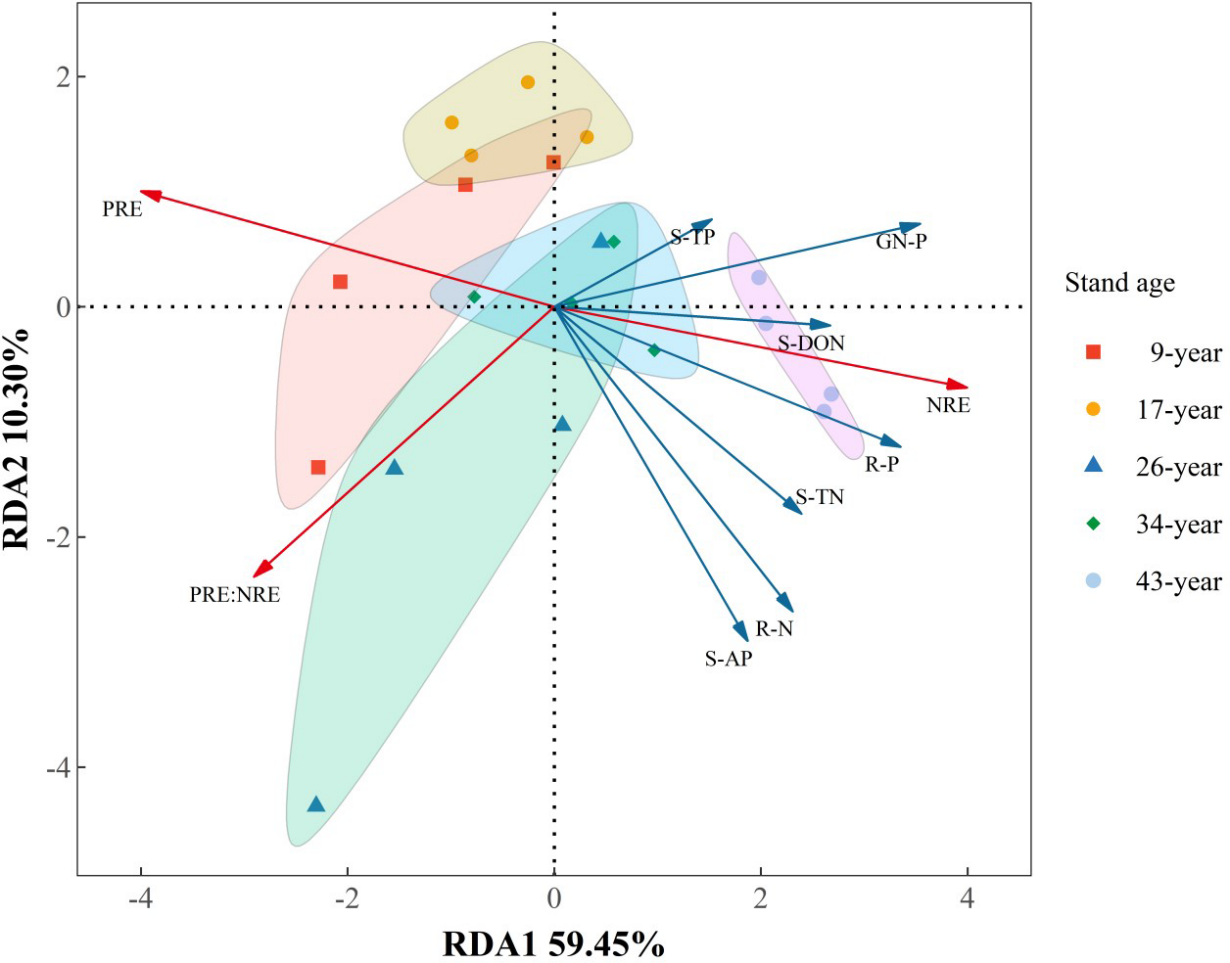
Redundancy analysis of the relationships between nutrient resorption attributes and nutrient status of soil and plant organs in *Pinus massoniana* plantations with different stand ages. PRE, phosphorus (P) resorption efficiency; NRE, nitrogen (P) resorption efficiency; S-TN, soil total N; S-TP, soil total P; S-DON, soil dissolved organic N; S-AP, soil available P; GN-P, Green needle P; R-N, root N; R-P, root P.

Alleviation of P limitation with increasing stand age of *P. massoniana* plantations was also supported by the relative portion of N and P resorbed during needle senescence. The “relative resorption hypothesis” stated that plants tend to resorb a greater portion of nutrient that limit their growth (Han et al., 2013), and PRE:NRE has been used to evaluate relative limitation of plant growth between N and P in forest ecosystems (Reed et al., 2012; Yan et al., 2018; Du et al., 2020). In this study, a negative correlation between PRE:NRE and stand age was observed, indicating that relatively less P was resorbed than N and P limitation was relatively decreased with increasing stand age. Moreover, a transition from PRE dominated to NRE dominated pattern of resorption with increasing stand age was also indicated by RDA analysis (**Figure 6**). This might be attributed to the different patterns of N and P resorption with stand age. According to Killingbeck (1996), N and P concentrations in senesced needles could be used to indicate complete or incomplete resorption by the living plant tissues. N was incomplete resorbed along the age sequence except for the 17-year-old stand, where a nearly complete resorption of N in the 17-year-old stand might be due to more N demand for biomass accumulation during the fast-growing period of *P. massoniana* (Zhou, 2001). Nevertheless, P concentrations in senesced needles indicated a shift from complete to incomplete resorption for P with increasing stand age, resulting higher concentrations of P and lower N:P in senesced needles in older stands (**Figures 2**, **3**). The lower senesced needle N:P in older stands could provide more P for microbes to synthesize ribosomal RNA and enhance the microbial metabolism of soil available P (Ren et al., 2016). Moreover, senesced needle C:N and C:P values were significantly lower in older stands than those in younger ones (**Figure 3**), indicating that senesced litterfall becomes easier to decompose and facilitates nutrient release with the stand development of *P. massoniana* plantations (William et al., 2007).

### Shifts in nutrient use strategy and nutrient resorption patterns with stand development

Plant nutrient-use strategy is generally identified based on green foliar nutrient content and nutrient resorption efficiency (Hayes et al., 2014). Plants from nutrient-poor site usually adopt “conservative consumption” nutrient-use strategy showing low nutrient concentration in green leaves and high leaf nutrient resorption during senescence, while at the nutrient-rich sites, plants usually have opposite characteristics and exhibit a “resource spending” nutrient-use strategy (Wright and Westoby, 2003). In this study, we found a shift from “conservative consumption” to “resource spending” P-use strategy with the stand development of *P. massoniana* plantations. The nutrient use strategy of a plant species could shift with changes in soil nutrient status during stand and successional development (Zeng et al., 2017; Wu et al., 2020). The shifts in P-use strategy were mainly attributed to the variations of soil P availability, as indicated by the results of RDA analysis and a significantly negative correlation between PRE and soil available P content (**Table 4**; **Figure 6**), combined with a marginally positive correlation between root P concentration and soil available P content, indicating that *P. massoniana* rely more on roots to acquire P rather than needle resorption. Moreover, fine root N/P did not change with stand development, but the N/P in the soil of the old stand was lower than in the fine roots, implying that more P taken up by fine roots. The change from conservative consumption to resource spending indicated a shift to more positive plant-soil feedback for P cycling with the stand development of *P. massoniana* plantations. In older stands with “resource spending” P-acquisition strategy, *P. massoniana* depends less on P resorption from senesced needles, and thus produces high-quality litters that are more easily decomposed. Therefore, the subsequent litter decomposition process returns more P from litter to soil, resulting in a positive feedback on the plant-soil P cycling.

Our results showed that the age-related nutrient-use strategy pattern varied with specific nutrient elements, which was consistent with the findings of previous studies (Zhang et al., 2018). Unlike P as discussed above, there was no clear pattern of needle N content and NRE of *P. massoniana* with stand age, and thus the N-use strategy. In consistent with previous study (Drenovsky et al., 2019), we observed that NRE and PRE respond differently to environment drivers such as soil nutrient availability. Since there was no relationship between NRE and soil N availability (**Table 3**), needle NRE of *P. massoniana* might be regulated by the interaction of soil N status and its eco-physiological characteristics rather than soil N availability alone. The highest NRE was observed in the fast-growing stage (17-year-old), since more N is needed to support the rapid biomass accumulation while root N storage capacity was still low (Zhou, 2001), *P. massoniana* depended more on needle resorption for N acquisition. There was no change observed in NRE across stand ages, despite an increase in soil N availability as stands grow old. The plasticity of N resorption was generally lower than that of P resorption (Xu et al., 2021). Moreover, higher N concentrations than P is required for plant growth and fitness (Hawkesford et al., 2012), the increase of N acquisition *via* root uptake (indicated by increased root N content with stand age) might not be enough for plant growth and development, therefore, a significant decreasing trend of NRE with stand age was not observed. The underlying mechanism needs to be further elucidated. In contrast, higher PRE was observed in younger stands under low soil P availability, which later decreased in older stands as soil P availability increased. Thus, reabsorption and soil nutrient availability was coupled for P but not for N (**Figure 6**), suggesting that besides soil N status internal N demand with age also controls reabsorption.

### Implications for forest plantation management

Nitrogen and phosphorus are two critical nutrients limiting tree growth in forest plantations (Hou et al., 2020; Zhang et al., 2022). Our results showed that the relative limitation status of N and P changed with stand age, with *P. massoniana* in young stands was co-limited by N and P, while the growth of *P. massoniana* in older stands was mainly limited by N. The decrease of P limitation with increasing stand age might be due to the increase of soil P availability. Thus, we suggested that fertilization management strategy in *P. massoniana* plantations should be modified with stand development, and increase the proportion of N fertilizer application in older stands. Moreover, mixed plantations with N fixing species are also suggested to alleviate N limitation in older *P. massoniana* stands, since the fixed N in plant materials would be slowly by steadily release to soil and then become available for *P. massoniana* (Lopez et al., 2014; Murata et al., 2022). Furthermore, our results showed that *P. massoniana* afforestation could improve soil fertility, especially soil P stock, which is the important limiting factor in subtropical soils. In addition to *P. massoniana*, *Cunninghamia lanceolata* is also an important afforestation species that are widely planted in subtropical China (State Forestry Administration of China, 2013). The planted area of *C. lanceolata* ranked first in China’s planted forest, however, land degradation and productivity decline has been observed in *C. lanceolata* plantations as a result of successive planting (Selvaraj et al., 2017; Liu et al., 2020; Guo et al., 2022). Wu et al. (2020) reported that a *C. lanceolata* plantation shifted from N limitation to P limitation with increasing stand age, while our results showed that P limitation decreased with stand development of *P. massoniana* plantation. Liu et al. (2016) showed that *P. massoniana* afforestation could improve soil quality in subtropical soil erosion areas of south China. Therefore, in order to alleviate the soil degradation caused by successive planting of *C. lanceolate*, *P. massoniana* seedlings are suggested as succession plantation at the *C. lanceolata* harvest site for the sustainable development of forest plantations in subtropical regions.

## Conclusion

PRE decreased while senesced needle P concentrations increased with stand age, indicating that there was a shift from “conservative consumption” to “resource spending” P-use strategy along the Masson pine plantation chronosequence. Higher green needle and root P concentration, as well as soil total and available P concentrations in older plantations were observed, along a decreasing trend of senesced needle C:P, N:P and PRE:NRE values with stand age, indicating that P limitation was alleviated with the stand development of *P. massoniana* plantations. Overall, our findings showed that *P. massoniana* is a useful afforested species to overcome ubiquitous P limitation in subtropical plantations. Our results provide novel information with important implications for the sustainable management of subtropical forest plantations.

## Data availability statement

The original contributions presented in the study are included in the article/supplementary material. Further inquiries can be directed to the corresponding author.

## Author contributions

YW and YH designed the project. WG, PJ and ZC conducted the experiments and analyzed the data. WG and YW wrote the paper. PJ, MLLC, LW, XL and YH reviewed and revised the manuscript. All authors contributed to the article and approved the submitted version.
